# On the Use of the Secale Cornutum in Lingering Labour

**Published:** 1826-04

**Authors:** Charles Waller

**Affiliations:** Surgeon.


ART. V.—*On the Use of the Secale Cornutum in lingering Labour.* By CHARLES WALLER, Surgeon.

NOTWITHSTANDING the mass of satisfactory information which has of late been accumulating with regard to the efficacy of the secale cornutum, in increasing the expulsatory efforts of the uterus, I find that a degree of scepticism still prevails on the subject; and therefore the following cases, which have occurred under my own observation, may not be uninteresting to your readers, as contributing to the general stock of knowledge which we already possess.

The two first cases in which I exhibited the remedy were, if my recollection serves me, (for I omitted to take notes of them,) very unsatisfactory, no apparent effect being produced by it; but in both the uterine action had nearly ceased, the pains being very trifling indeed, with long intervals between them. I employed the ergot in the way recommended by Dr. Merriman,—that is, in infusion: two drachms of secale cornutum, coarsely pulverised, and six ounces of boiling water poured upon it. After allowing it to stand twenty minutes, three ounces were given, and in the course of a quarter of an hour the remainder. I now resolved to increase the strength of the infusion for the future, using eight scruples instead of two drachms to six ounces of water; and the following case, which occurred on the 6th of April, 1825, convinced me that the remedy was one of considerable power.

CASE I.

I was sent for, about three o'clock in the morning, to Mrs. S—, ætat. twenty-six, a woman of delicate habit, who was in labour with her second child. I found the os uteri quite obliterated, and the external parts very dilatable, there being a copious mucous secretion. The pains were weak, and there appeared to be a considerable quantity of liquor amnii. I ruptured the membranes, and, upon examination, found the funis descending before the head. My patient was peculiarly anxious for a living child, the former one having been craniotomised. I pressed the cord beyond the head without difficulty, but it descended with the next pain. I then procured a piece of sponge, and intended to press the cord up beyond the head with it, and there leave it, in order that, by imbibing the moisture, it might so far enlarge as to prevent the descent of the funis; but the parts of generation were so tender, that the attempt gave her great pain: I therefore determined to desist, and to content myself by placing the cord in the most roomy part of the pelvis, as the head advanced. At nine o'clock, the pains appeared to be slightly increased, but the intervals between them were long; and, as I apprehended that the child's life depended upon its quick transit through the pelvis, I resolved to try the effect of the infusion of ergot, made in the proportion I have just mentioned, (eight scruples to six ounces of water.) I gave her three ounces at nine o'clock, (the head of the child was still above the brim,) and in less than half an hour the pains had increased to the utmost degree in intensity; in fact, I scarcely ever witnessed such violent uterine action. The child was born about ten o'clock, and, although at first feeble, it soon recovered, and, as well as the mother, did very well.

CASE II.

April, 1825, was requested by a gentleman in the city to visit a patient with him. She was thirty years of age, and was in labour with her first child. I saw her early in the morning, and was informed that she had been in labour all night. The pains were pretty regular, though not very strong; and, as the os uteri was not quite dilated, and the parts of generation still rigid, I recommended nothing to be done at present. The pains soon ceased altogether; but in the course of a few hours they were renewed, but so slightly as merely to produce a feeling of "forcing backwards," (to use the woman's own expression.) The parts being now more relaxed, I advised a trial of the infusion of ergot, which was given, (dose as above;) and, in about three-quarters of an hour, the pains became very strong, and continued so till the child was born. This patient lost a considerable quantity of blood after the expulsion of the placenta; the child did very well.

### CASE III.

In the month of April, 1825, Mrs. M—, ætat. about thirty, was taken with slight labour-pains, which continued during the whole of the night. Between nine and ten in the morning, finding that the pains did not increase, I resolved to try the infusion. The head was engaged in the pelvis, but the ear could not be felt. Three ounces of the infusion were administered, and, as it produced no effect, the dose was repeated in twenty minutes afterwards; and, in less than ten minutes from this time, the pains began to increase, and in about an hour the child was expelled. The child was livid in its appearance, and for the first minute or two did not breathe freely, there being scarcely a convulsive sob; but the circulation through the funis was very vigorous: it was therefore allowed to remain attached to the mother for a short time, and soon recovered.

I ought, perhaps, to state, that in this case the head was very large, and had consequently undergone a good deal of compression. It had, in a remarkable degree, that peculiar shape which, from its appearance, has been termed the sugar-loaf head. The mother did well.

### CASE IV.

May, 1825, Mrs. C—, a woman of plethoric habit, ætatis thirty, was taken in labour with her first child. I first saw her about noon, and found that the os uteri had scarcely began to open. At five in the afternoon, the waters came away; and, on examination, I found the os uteri about the size of a crown-piece, the remaining portion feeling as tough as a piece of the hardest leather: I never, either before or since, felt one in such a state. I took away about twelve ounces of blood, and ordered her an enema; after which, a dose of opium; waiting, however, a few hours, to see what effect the pains had upon the os uteri. By next morning, the child had entered the cavity of the pelvis, but did not advance, although she had tolerable pains. The arch of the pubis appeared rather narrower than usual, the rami approaching each other more closely than natural. I had only two drachms of ergot in the house, upon which I poured six ounces of boiling water, and gave it her at the usual intervals; thinking that, if I could succeed in producing an increase of the action of the uterus, the child might be born without having recourse to the use of an instrument. The medicine appeared to have but little effect, although I thought the pains were slightly increased; and in this opinion my friend, Mr. Chandler, of Staines, who was with me, concurred. It was found in this case necessary to deliver with the forceps, though, from the want of room from pubis to sacrum, I could not fix them in that situation, but was obliged to place them over the occiput and forehead. The perineum, however, was not injured by the instrument. The child was a very large one, and was born dead.

CASE V.

October, 1825, was sent for to Mrs. H—, ætat. twenty-nine, in labour with her third child. I first saw her about twelve at night. The pains were but trifling; the os uteri, however, considerably dilated. The membranes ruptured whilst I was there; the head was very high up, and, as the pains did not increase, I left her. She continued much in the same state all the day, and at five in the evening I gave her two of my usual doses of the infusion of ergot, and, about half an hour after the second draught, the pains were almost incessant, though not so violent as in some cases. At seven o'clock, I prepared a little decoction of ergot, made by boiling one drachm of the medicine in four ounces of water for about ten minutes, and directed one-third to be taken every hour. At half-past eight, the head had partly entered the brim; the patient became restless; the pains continued trifling, and there was a disposition to hemorrhage. Under these circumstances, Dr. Blundell was sent for; and, in consultation, it was agreed to deliver her with the long forceps. The child was born dead.

On examining her abdomen a few days after delivery, I found a considerable enlargement of the right ovarium, which had probably prevented the descent of the head. She had a considerable discharge of blood after delivery.

CASE VI.

October, 1825, saw Mrs. R—, ætat. twenty-eight, in labour with her second child. It was about four in the afternoon when I was first called, and, although she had pains at intervals, still the os uteri was not at all dilated. The liquor amnii was discharged at eight in the evening, when one of my pupils (Mr. Wylie) attended, and waited with her till eleven: finding the labour proceeding very slowly, he then left her. At half-past six the following morning, I was again summoned to the case. I found Mr. W. had been there since four. The os uteri was not completely dilated, but the remaining portion was so soft and yielding, that I apprehended it would afford no resistance, if the uterus could be made to act vigorously. The pains were at this time very trifling, and the intervals between them long. The patient was getting very irritable and fretful. I waited with her an hour, and then determined upon giving the ergot. The first dose (three ounces of the infusion) was given her at seven minutes before eight o'clock: the situation of the head at this time was partly above brim. Ten minutes after eight, the pains had certainly increased in frequency, though not in force. The infusion was now repeated; and, in about twelve minutes afterwards, it was truly gratifying to witness its effects. The pains were very severe, and returned every two and a half minutes, and a quarter before nine o'clock the head was expelled; the body of the child and the placenta soon followed.

This case was strikingly illustrative of the power which this remedy exerts over the action of the uterus: the increase of the pains was clearly owing to its exhibition; and, instead of being "few and far between," they were almost constant, and their force wonderfully increased. The female and child both did well.

### CASE VII.

This case was solely under the superintendance of Mr. Wylie. The patient had trifling pains for nearly thirty hours, the head above brim; she was getting irritable. My stock of ergot was reduced to one drachm, which Mr. W. infused in some boiling water, and gave her. The patient soon complained of an increase of pain, and in about an hour the child was born; which, as well as the mother, did well.

From the above cases, I am led to infer that the secale cornutum is a remedy which is capable of increasing the force of the uterine contractions in a most remarkable manner, under certain circumstances; but that the effect is doubtful, unless there be some degree of action present. In other words, that, although it will increase the contractions when already present, it will not always renew them when they are suspended.

That the effect is more certain if the infusion be of greater strength than is usually recommended; two drachms of the secale to six ounces of water being barely sufficient for the purpose.

That it appears to be a stimulus peculiarly fitted for irritable, and what are generally termed *nervous*, habits.

That the fears entertained by some practitioners of its proving detrimental to the child, are groundless.

But, although it is in general necessary, not only that thee should be a disposition for labour, but that this process should have actually commenced, before we can expect the secale conutum to have any effect upon the uterus, still one solitary case has indirectly come to my knowledge, (and I will vouch for the authenticity of it,) where this remedy was given for the purpose of producing abortion in a female about the second month of utero-gestation; and this effect was accomplished in a few hours after its exhibition.

111, *Aldersgate-street; March,* 1826.

